# Comparison of Disease Progression From Prostate Cancer Diagnosis to Metastatic or Nonmetastatic Castrate‐Resistant Prostate Cancer (CRPC) Patients: CaPA Study

**DOI:** 10.1002/cam4.71149

**Published:** 2025-08-20

**Authors:** Pedro Costa, António Patrício, João Vasco Barreira, Luís Abranches Monteiro, Luís Campos Pinheiro, Paulo Azinhais, Inês Sequeira, Carlos Rabaça, Ferdinando Pereira, Ricardo Borges, Francisco Botelho, Frederico Reis, José Carvalho, António Canelas, Hugo Coelho, Fernando Vila, Rui Dinis, Sílvia Dias, Ana Cristina Fialho, José Palma dos Reis

**Affiliations:** ^1^ Urology Department Vila Nova de Gaia Hospital Center Vila Nova de Gaia Portugal; ^2^ Urology Department Baixo Vouga Hospital Center Aveiro Portugal; ^3^ Oncology Department SAMS Lisbon Lisbon Portugal; ^4^ Urology Department Western Lisbon Hospital Center Lisbon Portugal; ^5^ Urology Department University Hospital Center of Central Lisbon Lisbon Portugal; ^6^ Urology Department Centro Hospitalar e Universitário de Coimbra Coimbra Portugal; ^7^ Oncology Department ULS Guarda Guarda Portugal; ^8^ Urology Department Instituto Português de Oncologia de Coimbra Francisco Gentil (IPO) Coimbra Portugal; ^9^ Urology Department SESARAM Funchal Portugal; ^10^ Urology Department Leiria Hospital Center Leiria Portugal; ^11^ Urology Department Centro Hospitalar de São João Oporto Portugal; ^12^ Urology Department ULS Matosinhos Matosinhos Portugal; ^13^ Urology Department Garcia da Orta Hospital Almada Portugal; ^14^ Urology Department Setúbal Hospital Center Setúbal Portugal; ^15^ Urology Department ULS Alto Minho – Viana do Castelo Viana do Castelo Portugal; ^16^ Urology Department Tâmega and Sousa Hospital Center Guilhufe Portugal; ^17^ Oncology Department Hospital Do Espírito Santo de Évora Évora Portugal; ^18^ Medical Department Janssen‐Cilag Farmacêutica, LDA Porto Salvo Portugal; ^19^ Urology Department Santa Maria Hospital Lisbon Portugal

**Keywords:** castrate‐resistant prostate cancer, disease management, prostate cancer, real‐world evidence, time to disease progression

## Abstract

**Background:**

Prostate cancer (PC) patients resistant to castration have decreased survival. Analysis of patient characteristics and disease management can contribute to new strategies to prevent or delay progression to castrate‐resistant prostate cancer (CRPC). This study aimed to characterize and compare PC patients from initial PC diagnosis to metastatic CRPC (mCRPC) versus nonmetastatic (nmCRPC) stages in a real‐world setting in Portugal.

**Methods:**

A multicenter, retrospective, non‐interventional study was conducted across 18 Portuguese sites. Consenting adult patients (age ≥ 18 years) diagnosed with CRPC, either metastatic or nonmetastatic, were included in the study at the time of diagnosis or within 12 months of CRPC diagnosis.

**Results:**

Between November 2020 and December 2022, a total of 121 patients (73 mCRPC patients and 48 nmCRPC patients) were included. At diagnosis, the median age was 69.0 and 70.0 years in mCRPC and nmCRPC patients, respectively, and the most common histological subtype was acinar adenocarcinoma (mCRPC: 83.6%; nmCRPC: 91.7%). Significant differences were observed between the two groups regarding Eastern Cooperative Oncology Group Performance status (ECOG PS) (*p* = 0.023), Gleason score (*p* = 0.001), and American Joint Committee on Cancer (AJCC) stage (*p* < 0.001). The median time from PC diagnosis to first treatment and the treatments used prior to CRPC diagnosis were comparable between the groups. However, the median time from PC diagnosis to CRPC was significantly shorter in mCRPC versus nmCRPC patients (42.0 vs. 58.0 months, *p* = 0.006). Prostate‐specific antigen (PSA) values were significantly higher in mCRPC than in nmCRPC patients at CRPC diagnosis (19.8 vs. 6.3 ng/mL; *p* < 0.001).

**Conclusions:**

This real‐world study showed that the time from initial PC diagnosis to castration resistance was significantly longer in nmCRPC than in mCRPC patients. The characteristics associated with a better prognosis at initial diagnosis and a better treatment response in nmCRPC patients might explain this difference in disease progression.

## 1 Introduction

Prostate cancer (PC) is the second leading cause of cancer‐related death in men [1, 2], and is considered a major health concern, with incidence rates varying across world regions [3, 4]. Differences in access to early diagnosis, PC management, and treatment [5] are reflected in the disease mortality rates.

PC is known to induce prostate‐specific antigen (PSA) release into the circulatory system, increasing its level in the blood. This serine protease has become a widely used marker for prostate cancer, employed in initial diagnosis, risk stratification, and monitoring of treatment response [6]. However, PSA levels should be considered in combination with other factors when deciding on disease management [6], such as the patient's age, health, and stage/extent of disease, including the presence or absence of metastasis (metastatic or nonmetastatic PC, respectively) [1].

Common treatment options include active surveillance, surgery, radiation therapy, chemotherapy or hormonal therapy, specifically androgen deprivation therapy (ADT) [7]. Moreover, although these approaches can be applied as single therapies, for certain men, a combination of different options may be indicated [7]. For instance, chemotherapy is usually combined with ADT in evidence of metastatic PC [8].

Active surveillance can be considered for patients diagnosed with low‐risk PC. It typically consists of periodical PSA testing, physical examinations, and prostate biopsies [9]. Radical prostatectomy and/or radiation therapy are effective treatments for men with more significant disease, namely those with higher PSA levels [7]. However, despite initial success with these treatments, up to a third of patients eventually develop biochemical recurrence characterized by a rising PSA [10].

Medical castration with ADT is considered a standard approach for prostate cancer treatment [11], based on the fact that the prostate is heavily reliant on androgens for growth and function. After the initiation of ADT, PSA levels in the blood almost always decrease and then stabilize for varying intervals [6]. Nevertheless, prostate cancer cells can become unresponsive, developing the ability to grow in androgen‐depleted conditions [11], and ultimately progressing to castration resistance.

Men with castrate‐resistant prostate cancer (CRPC) have a poor survival prognosis. Additionally, the time to castration resistance is a prognostic factor: patients who progress from initial PC diagnosis to CRPC in a shorter time have worse survival outcomes [12, 13].

Analyzing real‐world data on disease characteristics and management from initial PC diagnosis to the development of CRPC can help optimize disease management and prevent or, at least, slow disease progression to CRPC. Therefore, we aimed to characterize and compare the journey of PC patients from initial PC diagnosis to mCRPC or nmCRPC stages in terms of patient characteristics and disease management in the real‐world setting in Portugal.

## 2 Materials and Methods

### 2.1 Study Design and Patients

CaPA was a retrospective, multicenter, non‐interventional study designed to describe the journey from initial PC diagnosis to CRPC diagnosis in patients from 24 participant sites across Portugal (only 18 sites were able to enroll patients). The study was approved by the Independent Ethics Committees of the participating sites (see complete list in Table S1) and conducted in accordance with the Declaration of Helsinki and applicable national regulatory requirements.

Eligible patients were adult men aged 18 years or older with a confirmed diagnosis of CRPC, either metastatic or nonmetastatic, as defined by the Guidelines of the European Association of Urology (EAU) [14]. Patients were excluded if they were participating in clinical trials related to PC at the time of CRPC diagnosis. Enrollment occurred at the time of CRPC diagnosis or during subsequent follow‐up appointments, up to 12 months after CRPC onset. Informed consent was obtained from all patients prior to their inclusion in the study.

The following data were retrospectively collected, when available, from patients' medical records: demographics, medical history/relevant comorbidities, Eastern Cooperative Oncology Group performance status (ECOG PS) at initial PC diagnosis and at CRPC onset; PC‐related data including histological subtype, Gleason Score, American Joint Committee on Cancer (AJCC) stage, and PSA levels at PC and CRPC diagnosis; and treatments for PC management prior to CRPC diagnosis. PSA levels at various points between initial PC diagnosis and CRPC onset were also collected.

### 2.2 Statistical Analysis

The primary endpoint was the characterization of patient journey up to CRPC journey. Secondary endpoints included a comparison of the time from initial PC diagnosis to CRPC diagnosis, between patients with metastatic and nonmetastatic CRPC, as well as the evolution of PSA levels from initial PC diagnosis to CRPC diagnosis. The evaluation of variables was conducted on the full analysis set (FAS), which included all eligible patients. Study analyses were performed on two groups based on the metastatic status at the time of CRPC diagnosis: mCRPC versus nmCRPC patients.

Descriptive statistical analysis was performed for the study variables. The descriptive analysis included mean, median, standard deviation (SD), and interquartile range (IQR) for continuous variables, while categorical variables were described using counts and percentages. Continuous variables were compared using either the Mann–Whitney *U* test or Student's *t* test, and categorical variables were compared using the chi‐squared test or Fisher's exact test, as appropriate.

Kaplan–Meier analyses were conducted to estimate the time from initial PC diagnosis to either the first treatment for PC management or the onset of castration resistance. The log‐rank test was used to compare time‐to‐event curves between mCRPC and nmCRPC patients.

To assess the evolution of PSA values, descriptive analysis was used to compare PSA levels between the two groups (mCRPC vs nmCRPC patients) at different time points; an ANOVA model was applied to analyze PSA changes throughout the study in each group.

Bivariate and multivariate logistic regression analyses were performed to determine whether the following patient and disease characteristics at initial PC diagnosis were factors independently associated with the presence or absence of metastases at initial CRPC diagnosis: age, Gleason score, ECOG, and PSA level.

Missing data were not imputed and were left as missing. Statistical comparisons were conducted at a significance level of 0.05. Data analysis was performed using SAS version 9.4, under Enterprise Guide Interface v8.3.

## 3 Results

### 3.1 Patients

**FIGURE 1:**
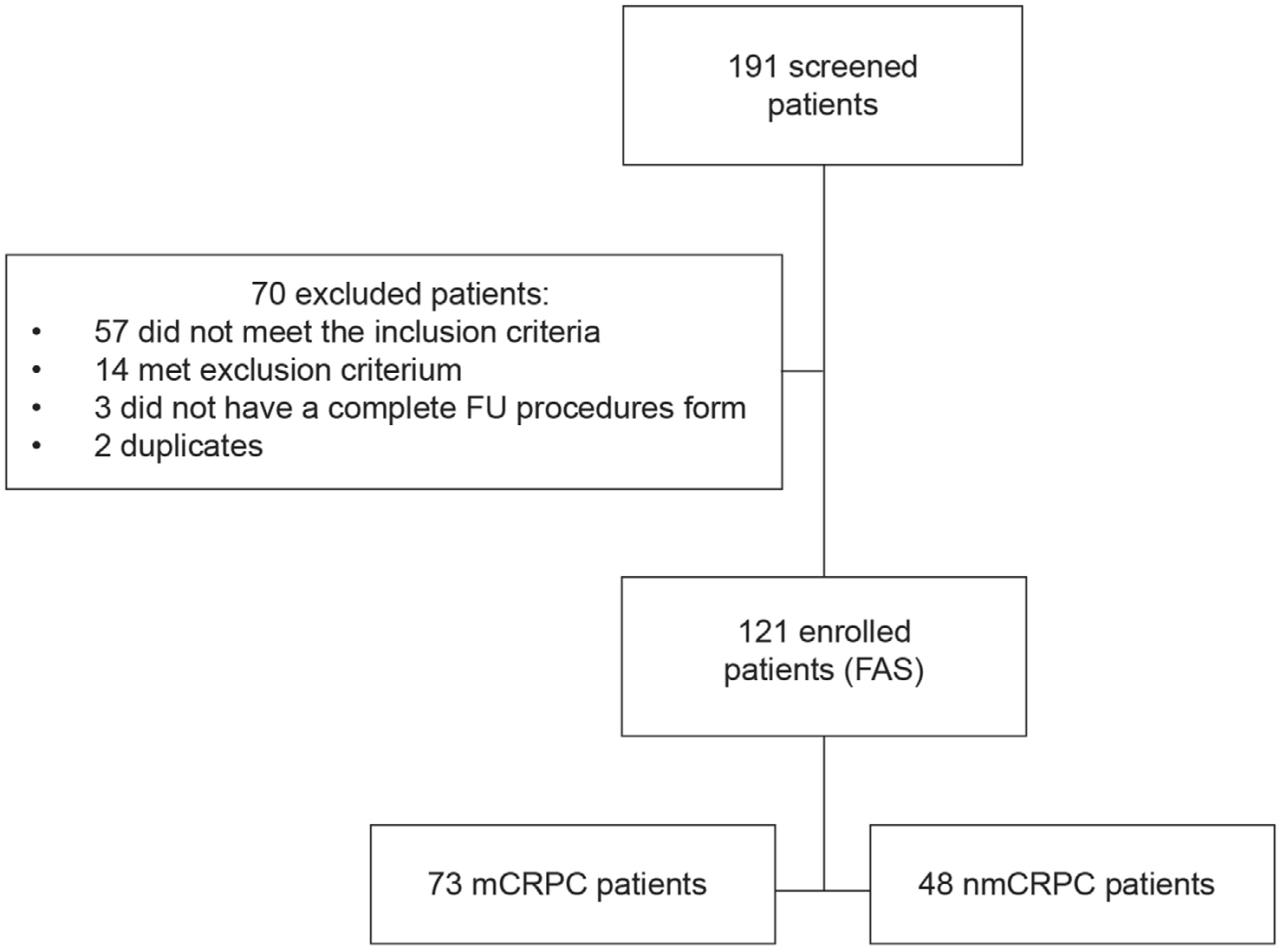
Summary of patient disposition. FAS, full analysis set; FU, follow‐up; mCRPC, metastatic castrate‐resistant prostate cancer; nmCRPC, nonmetastatic castrate‐resistant prostate cancer.

A total of 191 patients were screened between November 2020 and December 2022. Of those, 70 patients were excluded: 57 did not meet the inclusion criteria, 14 met exclusion criteria, three did not have complete follow‐up procedure forms, and two patients were duplicated. Consequently, 121 evaluable patients were included in the analysis and divided into two groups based on metastatic status at CRPC diagnosis: mCRPC patients (*n* = 73) and nmCRPC patients (*n* = 48) (Figure 1). The list of participants and the number of enrolled patients per site are provided in Table S1.

### 3.2 Prostate Cancer Diagnosis

At the time of initial PC diagnosis, the median age of patients was 69 and 70 years for mCRPC and nmCRPC patients, respectively (Table 1). The two groups showed a significant difference in ECOG PS (*p* = 0.023), with 82.3% of mCRPC patients and 88.6% of nmCRPC patients having an ECOG PS of 0. Significant differences were also observed between mCRPC and nmCRPC groups regarding the histological subtype at PC diagnosis (*p* = 0.039). Acinar adenocarcinoma was the most common subtype in both groups (mCRPC: 83.6%; nmCRPC: 91.7%). Gleason scores (primary score) significantly differed between groups (*p* = 0.001): 16.7% of mCRPC and 33.3% of nmCRPC patients had a Gleason score of 3; 65.3% and 50.0%, respectively, had a score of 4; and 6.9% and 8.3%, respectively, had a score of 5. At PC diagnosis, most patients were in stage IV (Table 1).

**TABLE 1:** Patient and disease characteristics at initial PC diagnosis and CRPC onset.

	Initial PC diagnosis			CRPC diagnosis		
	mCRPC	nmCRPC	*p*	mCRPC	nmCRPC	*p*
Age^a^ (years)			0.942			0.382
*N*	73	48				
Median (IQR)	69.0 (62.0–76.0)	70.0 (62.5–77.5)		76.0 (71.0–82.0)	79.0 (70.5–86.0)	
ECOG PS, *n* (%)			**0.023**			**0.006**
*N*	62	44		61	41	
ECOG 0	51 (82.3%)	39 (88.6%)		36 (59.0%)	24 (58.5%)	
ECOG 1	8 (12.9%)	3 (6.8%)		16 (26.2%)	13 (31.7%)	
ECOG 2	3 (4.8%)	1 (2.3%)		6 (9.8%)	1 (2.4%)	
ECOG 3	0 (0.0%)	1 (2.3%)		3 (4.9%)	3 (7.3%)	
Comorbidities^b^, *n* (%)						
*N*	73	48				
Diabetes	15 (20.6%)	14 (29.2%)	0.277	NA	NA	
Cardiovascular disease						
Heart failure	3 (4.1%)	3 (6.2%)	0.681	NA	NA	
Others	23 (31.5%)	19 (39.6%)	0.361	NA	NA	
Histological subtype, *n* (%)			**0.039**			**0.040**
*N*	73	48		73	48	
Acinar adenocarcinoma	61 (83.6%)	44 (91.7%)		58 (79.4%)	39 (81.2%)	
Prostatic intraepithelial neoplasia	1 (1.4%)	0 (0.0%)		0 (0.0%)	0 (0.0%)	
Ductal adenocarcinoma	1 (1.4%)	0 (0.0%)		1 (1.4)	0 (0.0%)	
Metastatic tumor	1 (1.4%)	0 (0.0%)		4 (5.5%)	2 (4.2%)	
Not available/unknown	9 (12.3%)	4 (8.3%)		10 (13.7%)	7 (14.6%)	
Gleason score (primary score), *n* (%)			**0.001**			0.071
*N*	72	48		73	48	
3	12 (16.7%)	16 (33.3%)		8 (11.0%)	14 (29.2%)	
4	47 (65.3%)	24 (50.0%)		47 (64.4%)	22 (45.8%)	
5	5 (6.9%)	4 (8.3%)		6 (8.2%)	4 (8.3%)	
Not applicable	8 (11.1%)	4 (8.3%)		12 (16.4%)	8 (16.7%)	
Gleason score (secondary score), *n* (%)		0.301			0.402	
*N*	72	48		73	48	
3	17 (23.6%)	16 (33.3%)		16 (21.9%)	14 (29.2)	
4	31 (43.1%)	23 (47.9%)		27 (37.0%)	20 (41.7%)	
5	16 (22.2%)	5 (10.4%)		18 (24.7%)	6 (12.5%)	
Not applicable	8 (11.1%)	4 (8.3%)		12 (16.4%)	8 (16.7%)	
AJCC stage, *n* (%)			**< 0.001**		**< 0.001**	
*N*	35	9		73	8	
IIIA	1 (2.9%)	0 (0.0%)		0 (0.0%)	2 (25.0%)	
IVA	0 (0.0%)	6 (66.7%)		0 (0.0%)	6 (75.0%)	
IVB	34 (97.1%)	3 (33.3%)		73 (100.0%)	0 (0.0%)	

*Note:* Bold indicates statistical significance (*p* < 0.05).

Abbreviations: AJCC, American Joint Committee on Cancer; ECOG PS, Eastern Cooperative Group performance status; IQR, interquartile range; NA, Not available.

^a^ Age at the inclusion in the study, which could be either at CRPC diagnosis or in subsequent appointments, up to a maximum of 12 months after the moment of CRPC diagnosis.

^b^ Comorbidities in more than 20% of patients in each group: diabetes and cardiovascular disease.

### 3.3 Therapeutic Management of PC

**FIGURE 2:**
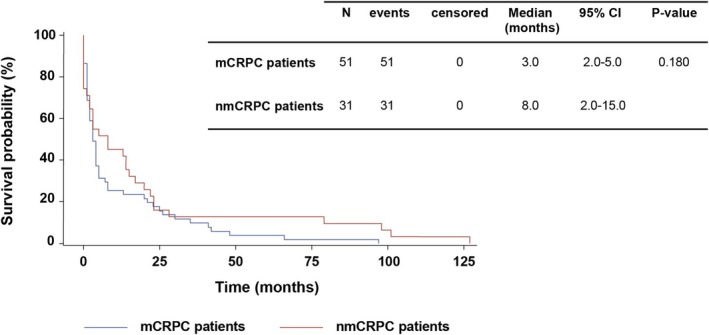
Kaplan–Meier estimates of time from PC diagnosis to the first treatment.

The median time to initiation of treatment for PC management was < 1 year after PC diagnosis. Although nmCRPC patients started treatment at a median time more than twice as long as mCRPC patients, the difference was not statistically significant (Figure 2).

Regarding the treatments, overall, 20 (27.4%) mCRPC patients and 8 (16.7%) nmCRPC patients underwent surgery, while 28 (38.4%) and 21 (43.8%), respectively, received radiotherapy, without significant differences in the types of surgery or radiotherapy used between the groups (Table 2). Similarly, no significant differences were observed in the types of hormonal therapy used. Pharmacological castration was employed for PC treatment in 93.4% of patients (mCRPC: 93.2%; nmCRPC: 93.8%). Luteinizing hormone‐releasing hormone (LHRH) agonists were administered in 82.6% of patients (mCRPC: 83.6%; nmCRPC: 81.3%), and antiandrogens were used in 58.7% of patients (mCRPC: 57.5%; nmCRPC: 60.4%). Chemotherapy with docetaxel was administered exclusively to 9 (12.3%) mCRPC patients.

**TABLE 2:** Treatments for PC management prior to CRPC diagnosis.

	mCRPC (*N* = 73)	nmCRPC (*N* = 48)	*p*
**Surgery, *n* (%)**	20 (27.4%)	8 (16.7%)	
Radical prostatectomy^a^	12 (16.4%)	7 (14.6%)	0.784
Orchiectomy^a^	5 (6.8%)	1 (2.1%)	0.401
Pelvic lymph node dissection^a^	5 (6.8%)	0 (0.0%)	0.156
**Radiotherapy, *n* (%)**	28 (38.4%)	21 (43.8%)	
External beam radiation therapy^a^	18 (24.7%)	17 (35.4%)	0.202
Proton beam radiation therapy^a^	1 (1.4%)	1 (2.1%)	1.000
LDR brachytherapy^a^	3 (4.1%)	2 (4.2%)	1.000
HDR brachytherapy^a^	3 (4.1%)	1 (2.1%)	1.000
**Pharmacological castration, *n* (%)**	68 (93.2%)	45 (93.8%)	
LHRH agonists^a^	61 (83.6%)	39 (81.3%)	0.743
LHRH antagonists^a^	17 (23.3%)	10 (20.8%)	0.751
Antiandrogens^a^	42 (57.5%)	29 (60.4%)	0.753
Steroidal anti‐androgens^a^	6 (8.2%)	6 (12.5%)	0.538
Nonsteroidal anti‐androgens^a^	40 (54.8%)	27 (56.2%)	0.875
**Chemotherapy, *n* (%)**	9 (12.3%)	0 (0.0%)	
Docetaxel^a^	9 (12.3%)	0 (0.0%)	**0.011**

*Note:* Bold indicates statistical significance (*p* < 0.05).

Abbreviations: HDR, high‐dose‐rate; LDR, low dose rate; LHRH, Luteinizing Hormone‐Releasing Hormone.

^a^ Number of patients who received the treatment at least once.

Treatment response differed significantly between mCRPC and nmCRPC patients for external beam radiation therapy, LHRH agonists and antagonists, and steroidal and nonsteroidal antiandrogens. The objective response rate (ORR) was higher in nmCRPC patients compared to mCRPC patients for all treatments used in PC management, except for nonsteroidal antiandrogens. A higher rate of complete response was also observed in nmCRPC patients compared to mCRPC patients (Table 3).

**TABLE 3:** Treatment response.

	mCRPC	nmCRPC	*p*
**Radiotherapy** ^a^			
External beam radiation therapy, *n* (%)			**0.015**
*N*	15	12	
Complete response	4 (26.7%)	5 (41.7%)	
Partial response	4 (26.7%)	3 (25.0%)	
Stable disease	5 (33.3%)	1 (8.3%)	
Progression	2 (13.3%)	3 (25.0%)	
**Pharmacological castration**			
LHRH agonists, *n* (%)			**0.002**
*N*	59	38	
Complete response	3 (5.1%)	5 (13.2%)	
Partial response	18 (30.5%)	15 (39.5%)	
Stable disease	15 (25.4%)	7 (18.4%)	
Progression	23 (39.0%)	11 (29.0%)	
LHRH antagonists, *n* (%)			**0.018**
*N*	19	9	
Complete response	0 (0.0%)	0 (0.0%)	
Partial response	11 (57.9%)	6 (66.7%)	
Stable disease	2 (10.5%)	3 (33.3%)	
Progression	6 (31.6%)	0 (0.0%)	
Steroidal anti‐androgens, *n* (%)			**0.016**
*N*	5	5	
Complete response	0 (0.0%)	1 (20.0%)	
Partial response	0 (0.0%)	3 (60.0%)	
Stable disease	2 (40.0%)	0 (0.0%)	
Progression	3 (60.0%)	1 (20.0%)	
Nonsteroidal anti‐androgens, *n* (%)			**0.001**
*N*	44	27	
Complete response	1 (2.3%)	3 (11.1%)	
Partial response	17 (38.6%)	5 (18.5%)	
Stable disease	10 (22.7%)	4 (14.8%)	
Progression	16 (36.4%)	15 (55.6%)	
**Chemotherapy**			
Docetaxel, *n* (%)			Not applicable
*N*	9	0	
Complete response	1 (11.1%)	0 (0.0%)	
Partial response	4 (44.4%)	0 (0.0%)	
Stable disease	4 (44.4%)	0 (0.0%)	
Progression	0 (0.00)	0 (0.0%)	

*Note:* Bold indicates statistical significance (*p* < 0.05).

^a^ It includes only data from radiotherapy techniques with a minimum sample size of five patients.

### 3.4 Castration‐Resistant Prostate Cancer Diagnosis

**FIGURE 3:**
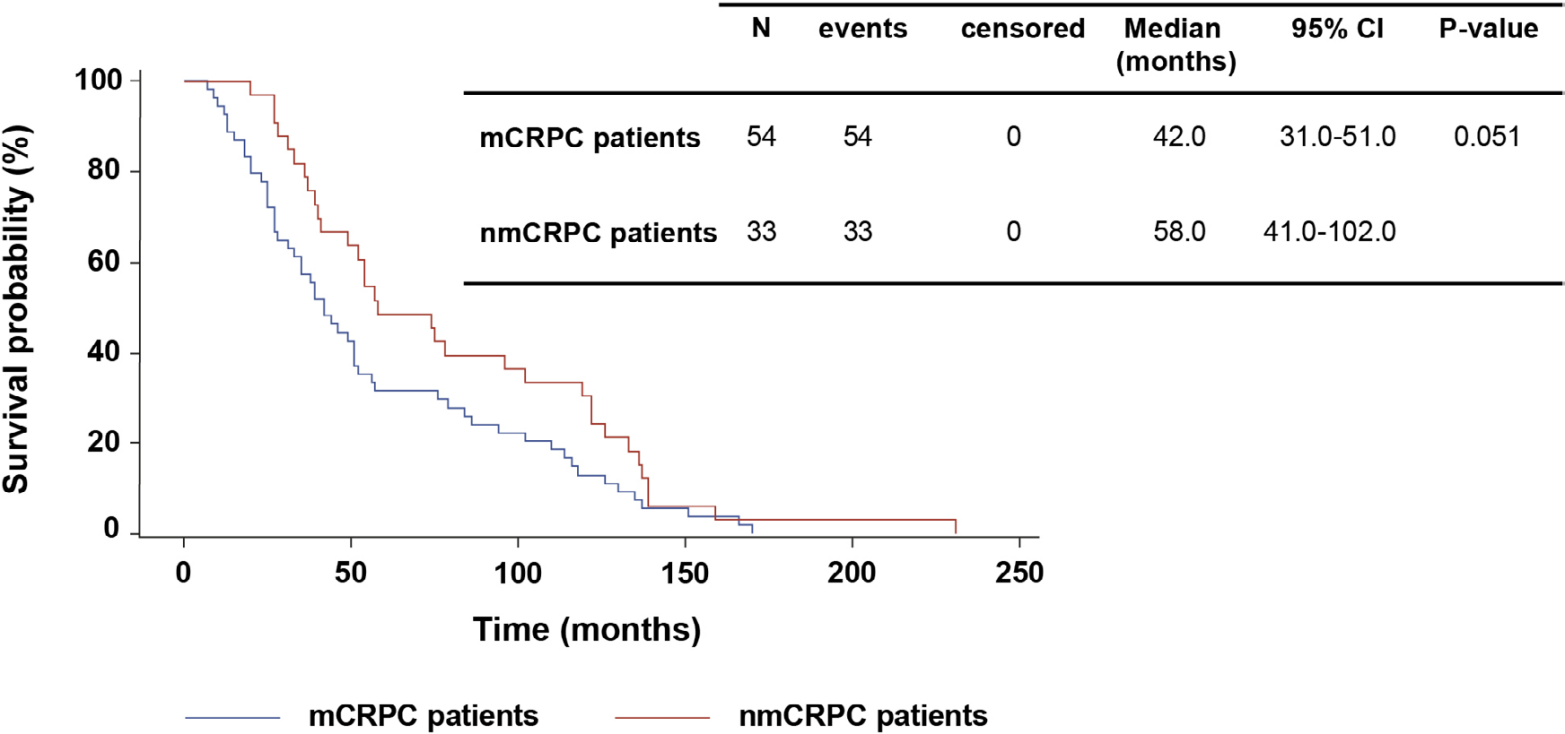
Kaplan–Meier estimates of time from PC diagnosis to CRPC diagnosis.

The median time from initial PC diagnosis to CRPC onset was significantly shorter in mCRPC compared to nmCRPC patients, as determined by the descriptive analysis using Mann–Whitney's *U* test: 42.0 [25.0–86.0] vs. 58.0 [39.0–122.0] months, respectively (*p* = 0.006). Kaplan–Meier estimates confirmed the same median times due to the absence of censored events but showed differing interquartile ranges, with a *p*‐value that approached statistical significance (Figure 3).

Patients' ECOG PS declined from initial PC diagnosis to CRPC diagnosis in both groups. However, at CRPC diagnosis, ECOG PS significantly differed between the groups, with a higher percentage of nmCRPC patients maintaining an ECOG PS of 0–1 (Table 1). The Gleason score showed no significant differences between the two groups.

### 3.5 PSA Levels Evolution

**FIGURE 4:**
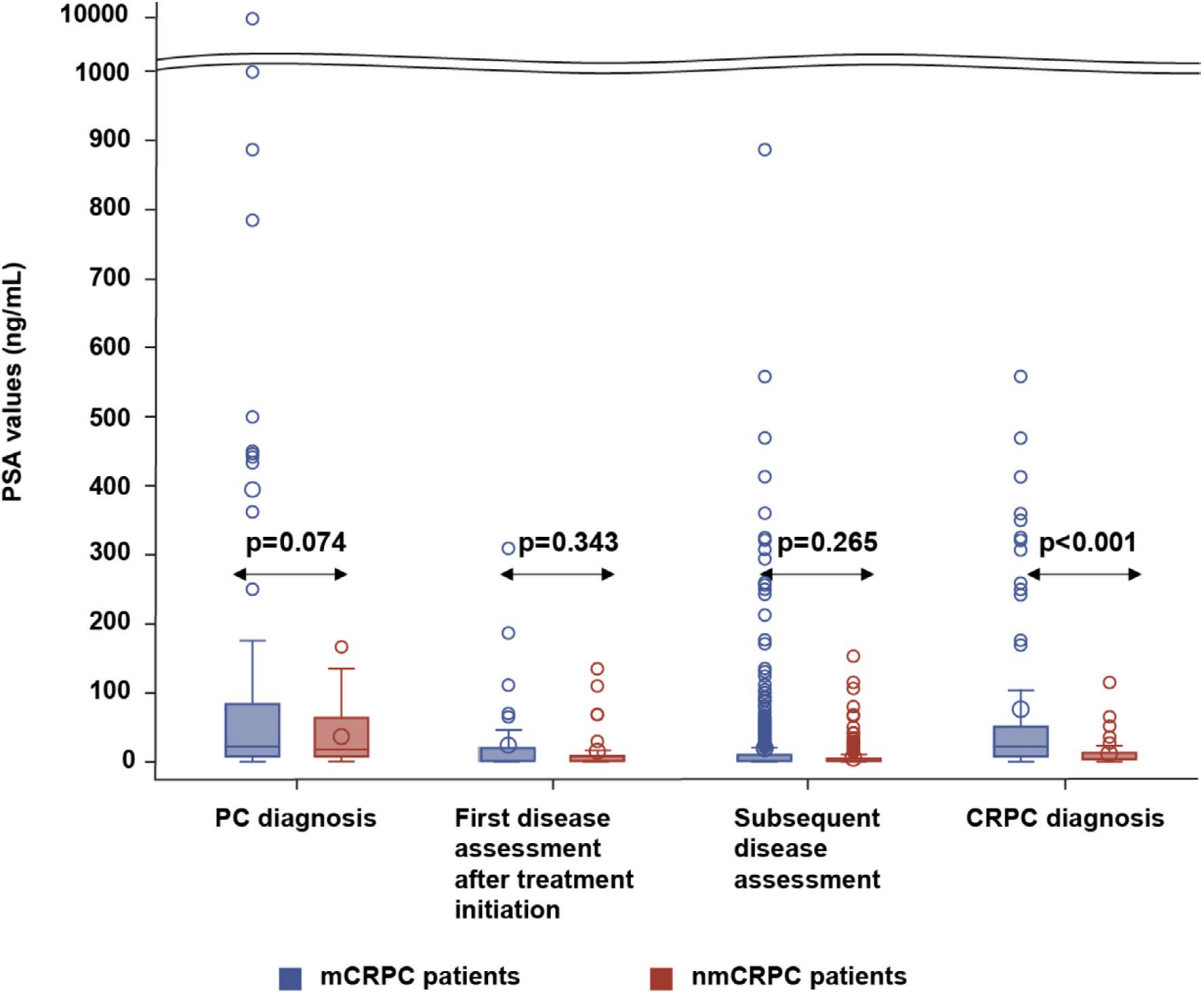
PSA levels evolution in mCRPC and nmCRPC patients.

The comparison of PSA levels between mCRPC and nmCRPC patients at various time points (from initial PC diagnosis to CRPC diagnosis) revealed a significant difference only at CRPC diagnosis (Figure 4). The median PSA level at CRPC diagnosis was 19.8 ng/mL for mCRPC patients and 6.3 ng/mL for nmCRPC patients (*p* < 0.001). Analysis of variance for PSA levels was not significant in mCRPC patients (*p* = 0.178), but was significant in nmCRPC patients (*p* = 0.006). Among nmCRPC patients, a significant reduction in PSA levels was observed during both the first and subsequent assessments following the initiation of treatment, compared to PSA levels at the initial PC diagnosis (Table 4).

**TABLE 4:** Change in PSA levels between different time points in nmCRPC patients.

	nmCRPC	
	*N* = 24	*p*
**From PC diagnosis to 1st disease assessment after treatment initiation**		
Median (IQR)	−8.1 (−36.4 to −1.4)	**0.016**
**From 1st disease assessment after treatment initiation to subsequent disease assessment**		
Median (IQR)	0.3 (−1.3 to 1.5)	0.692
**From PC diagnosis to subsequent disease assessment**		
Median (IQR)	−7.8 (−34.3 to −4.8)	**0.009**
**From PC diagnosis to CRPC diagnosis**		
Median (IQR)	−5.7 (−24.1 to 1.0)	0.223
**From 1st disease assessment after treatment initiation to CRPC diagnosis**		
Median (IQR)	4.0 (2.3 to 10.4)	0.692
**From subsequent disease assessment to CRPC diagnosis**		
Median (IQR)	3.8 (1.8 to 11.7)	0.692

*Note:* Bold indicates statistical significance (*p* < 0.05).

Abbreviation: IQR, interquartile range.

### 3.6 Risk Factors for the Development of Metastases at CRPC Diagnosis

Age, Gleason score, ECOG PS, and PSA values at PC diagnosis were not associated with the metastatic status of patients at CRPC diagnosis, as determined by both bivariate and multivariate analyses (Table 5).

**TABLE 5:** Bivariate and multivariate analysis of risk factors associated with metastatic status at CRPC diagnosis.

Risk factor	Bivariate analysis			Multivariate analysis		
	OR	95% CI	*p*	OR	95% CI	*p*
Age^a^	1.013	0.968–1.060	0.585	1.016	0.964–1.071	0.554
ECOG PS						
1	0.653	0.153–2.789	0.964	0.915	0.181–4.636	0.968
2	0.653	0.057–7.485	0.964	0.583	0.044–7.714	0.962
3	> 999.999	< 0.001–> 999.999	0.966	> 999.999	< 0.001–> 999.999	0.966
Gleason primary score (referral category: score 5)						
3	1.875	0.402–8.738	0.134	2.018	0.404–10.087	0.164
4	0.688	0.167–2.827	0.146	0.799	0.183–3.488	0.260
Gleason secondary score (referral category: score 5)						
3	2.427	0.687–8.578	0.369	1.557	0.393–6.158	0.602
4	2.477	0.773–7.942	0.282	1.401	0.394–4.981	0.805
PSA Value	0.996	0.990–1.002	0.174	0.997	0.992–1.003	0.296

Abbreviations: ECOG PS, Eastern Cooperative Group performance status; OR, odds ratio.

^a^ Age at the inclusion in the study (either at CRPC diagnosis or in subsequent appointments, up to a maximum of 12 months after the moment of CRPC diagnosis).

## 4 Discussion

At PC initial diagnosis, the two groups of patients (divided into mCRPC patients and nmCRPC patients based on their metastatic status at CRPC diagnosis) had a balanced population in terms of age and comorbidities. Both groups had a median age above 65 years consistent with the known fact that the highest incidence occurs in elderly men [15, 16]. Additionally, acinar adenocarcinoma was the most common histological subtype, as observed globally [17].

In our study, analysis of the patients' journey from initial PC diagnosis to CRPC showed that neither the time to start the first treatment nor the treatments used (except for docetaxel) differed between mCRPC and nmCRPC patients, suggesting similar disease management for both groups. However, the median time from initial PC diagnosis to CRPC onset was significantly longer for nmCRPC patients than for mCRPC patients in the descriptive analysis using the Mann–Whitney *U* test. In the Kaplan–Meier method, the median time from initial PC diagnosis to CRPC diagnosis was on the edge of statistical significance. Still, the survival curves of the two groups remained separated for more than 100 months, indicating a clinically meaningful result [18, 19].

Two non‐mutually exclusive hypotheses may explain the extended time from initial PC diagnosis to CRPC diagnosis in the nmCRPC group. On one hand, nmCRPC patients generally had a better prognosis than mCRPC patients based on baseline patient and disease characteristics at initial PC diagnosis. This included a higher frequency of patients with ECOG 0 and Gleason score (primary score) 3 in the nmCRPC group compared to the mCRPC group. The statistically significant difference between the two groups regarding the AJCC stage should be interpreted with caution due to the small sample size analyzed in the nmCRPC group. Nonetheless, the data presented here indicate that most patients who later developed mCRPC had advanced, metastatic disease at initial PC diagnosis. The high proportion of stage IVB patients at initial diagnosis in the mCRPC group also highlights the predictive value of initial staging, suggesting that early detection and intervention may be crucial in preventing or delaying the development of metastatic disease. On the other hand, although treatment options were generally similar between the groups, the ORR was higher for nmCRPC patients than for mCRPC patients for all treatments described above, except for nonsteroidal antiandrogen. Nevertheless, a higher percentage of nmCRPC patients achieved complete response compared to mCRPC patients. In addition, only the nmCRPC patients showed a significant decrease in PSA levels between the initial PC diagnosis and different disease assessment points after treatment initiation.

A better prognosis, a better treatment response, and/or a significant decrease in PSA values after treatment initiation may have played a role in delaying the progression to CRPC in the nmCRPC group. Nonetheless, this interpretation must be approached with caution due to the small sample size of some analyzed parameters, especially regarding treatment response. Furthermore, we cannot rule out the possibility of inconsistencies in the timing of monitoring procedures and care visits between patients in the two groups after the start of the first treatment, which could also have contributed to the different outcomes observed.

A previous Japanese study, which aimed to compare the overall survival of nmCRPC versus mCRPC patients under a treatment used after progression to CRPC, showed a longer median time to CRPC in the nmCRPC group than in the mCRPC group, with a *p*‐value at the threshold of statistical significance: 42.0 (0.7–138.0) versus 28.3 (4.0–140.0); *p* = 0.05 [20]. This study, conducted at a single site with an Asian population and a small sample size, faced limitations in the power of its statistical analysis. In contrast, our study performed in a European population with a larger sample size and with its multicentric nature encompassing geographic diversity (including sites across rural and urban areas) showed not only a statistically significant longer time to CRPC for nmCRPC patients compared to mCRPC patients in the descriptive analysis but also a clinically meaningful difference highlighted by the Kaplan–Meier survival curves. Thus, our meaningful results indicate the generalizability of the evidence that nmCRPC patients progress to CRPC later than mCRPC patients.

Unlike the Japanese retrospective study, we did not analyze the overall survival upon a treatment for CRPC. However, the fact that, at the time of CRPC diagnosis, the nmCRPC group continued to exhibit disease and patient characteristics associated with a better prognosis than the mCRPC group, including a significantly lower PSA value [21]—which is a known predictor of survival [22]—suggests a potentially superior survival outcome for patients with nmCRPC, as observed in the Japanese study [20].

As the presence of metastases in patients diagnosed with CRPC is a poor prognostic factor of survival [23], we aimed to identify possible associations between variables routinely assessed at initial PC diagnosis—age, Gleason score, ECOG PS, PSA level—and the development and presence of metastases at the time of CRPC diagnosis. Our goal was to identify factors that could predict patients at a high risk of developing metastases to enable anticipation and adjustment of treatment based on risk stratification. Both the bivariate and multivariate logistic regression analyses failed to find an association between any of these variables and metastatic status at CRPC diagnosis. These data suggest that those variables cannot be used as predictive markers for the development of metastases. Nevertheless, a study with a larger sample size is necessary to confirm these results.

The CaPA study has the strength of being a multicenter study that included sites across the country, encompassing both rural and urban areas. However, it has also limitations. One inherent limitation of observational retrospective studies is the lack of data at participating centers. Additionally, patients who died of CRPC before disease assessment and CRPC diagnosis were not included in the study. Lastly, the power of the analysis was limited because only 121 of the planned 300 patients were included. Despite these limitations, the study provided valuable insights into the delayed progression from initial PC diagnosis to nmCRPC compared to progression to mCRPC, as well as into the significant variance in PSA levels after treatment initiation, which was observed only in nmCRPC patients.

Factors that can accurately predict high‐risk patients will play a crucial role in treatment selection and in the investigation of mechanisms to prevent or delay the development of CRPC and/or metastases throughout the disease. Hence, although our findings require confirmation in a larger sample study, they appear to have the potential to help improve the management of patients with PC.

## 5 Conclusion

To our knowledge, this was the first real‐world study to characterize and compare the journey of PC patients from initial diagnosis to mCRPC and nmCRPC stages in terms of patients' characteristics and disease management in the real‐world setting in Portugal.

While recent studies have shown that the longer the time to castration resistance in metastatic hormone‐sensitive PC patients, the better their overall survival [12, 13], our study further highlighted that time to castration resistance is a distinguishing characteristic between nmCRPC and mCRPC patients.

Nevertheless, a study with a larger sample size will be necessary to test and validate whether an overall better prognosis, a better response to treatment, and/or a significant reduction of the PSA levels between different time points of disease assessment after treatment initiation and PC diagnosis explain the longer time to castration resistance in patients diagnosed with nmCRPC versus mCRPC. Identifying the factors responsible for a shorter versus longer time to castration resistance may improve PC management and lead to the design of new studies aimed at preventing or delaying progression to CRPC.

## Author Contributions

**Pedro Costa:** investigation (lead), writing – original draft (lead), writing – review and editing (equal). **António Patrício:** investigation (equal), writing – original draft (equal), writing – review and editing (equal). **João Vasco Barreira:** investigation (equal), writing – original draft (equal), writing – review and editing (equal). **Luís Abranches Monteiro:** investigation (equal), writing – original draft (equal), writing – review and editing (supporting). **Luís Campos Pinheiro:** investigation (equal), writing – original draft (equal), writing – review and editing (supporting). **Paulo Azinhais:** investigation (equal), writing – original draft (equal), writing – review and editing (supporting). **Inês Sequeira:** investigation (equal), writing – original draft (equal), writing – review and editing (supporting). **Carlos Rabaça:** investigation (equal), writing – original draft (equal), writing – review and editing (supporting). **Ferdinando Pereira:** investigation (equal), writing – original draft (equal), writing – review and editing (supporting). **Ricardo Borges:** investigation (equal), writing – original draft (equal), writing – review and editing (supporting). **Francisco Botelho:** investigation (equal), writing – original draft (equal), writing – review and editing (supporting). **Frederico Reis:** investigation (equal), writing – original draft (equal), writing – review and editing (supporting). **José Carvalho:** investigation (equal), writing – original draft (equal), writing – review and editing (supporting). **António Canelas:** investigation (equal), writing – original draft (equal), writing – review and editing (supporting). **Hugo Coelho:** investigation (equal), writing – original draft (equal), writing – review and editing (supporting). **Fernando Vila:** investigation (equal), writing – original draft (equal), writing – review and editing (supporting). **Rui Dinis:** investigation (equal), writing – original draft (equal), writing – review and editing (supporting). **Sílvia Dias:** project administration (equal), writing – original draft (equal), writing – review and editing (supporting). **Ana Cristina Fialho:** conceptualization (equal), data curation (supporting), formal analysis (supporting), methodology (lead), project administration (equal), writing – original draft (supporting), writing – review and editing (supporting). **José Palma dos Reis:** conceptualization (lead), data curation (supporting), formal analysis (supporting), writing – original draft (lead), writing – review and editing (lead).

## Consent

All enrolled patients provided written informed consent.

## Conflicts of Interest

A.P. has participated on advisory boards for Janssen‐Cilag Farmacêutica, Lda; A.C.F. and S.D. are employees of Janssen‐Cilag Farmacêutica, Lda; F.B. has participated as a consultant or speaker for Astellas, Janssen‐Cilag Farmacêutica, Lda, and Bayer. J.P.R. has participated in lectures subject to honoraria and advisory boards for Janssen‐Cilag Farmacêutica, Lda, Bayer, and Astellas; and I.S. has participated as a consultant for Ipsen and Astellas and was sponsored to go to conferences by Janssen‐Cilag Farmacêutica, Lda, MSD, and Pfizer. P.C., J.V.B., L.A.M., L.C.P., P.A., C.R., F.P., R.B., F.R., J.C., A.C., H.C., F.V., and R.D. declare no conflicts of interest.

## Supporting information


**Table S1:** Participant sites, number of enrolled patients and Independent Ethics Committees (IECs).

## Data Availability

The data that support the findings of this study are available on request from the corresponding author. The data are not publicly available due to privacy or ethical restrictions.
