# Supplementary material for: Comparison of Disease Progression From Prostate Cancer Diagnosis to Metastatic or Nonmetastatic Castrate‐Resistant Prostate Cancer (CRPC) Patients: CaPA Study
Source: Cancer Med. 2025 Aug 20;14(16):e71149. doi: 10.1002/cam4.71149 (PMC12365608; doi:10.1002/cam4.71149)
Supplement: Supplementary file 1 — Table S1: Participant sites, number of enrolled patients and Independent Ethics Committees (IECs). [file CAM4-14-e71149-s001.docx]

**Table S1.** Participant sites, number of enrolled patients and Independent Ethics Committees (IECs).

|  | Sites | Enrolled patients | IECs |
| --- | --- | --- | --- |
| 1 | Vila Nova de Gaia Hospital Center | 22 | Comissão de Ética para a Saúde do Centro Hospitalar de Vila Nova de Gaia/Espinho, EPE |
| 2 | Baixo Vouga Hospital Center | 17 | Comissão de Ética para a Saúde do Centro Hospitalar Baixo Vouga, EPE |
| 3 | Santa Maria Hospital | 12 | Comissão de Ética do Centro Académico de Medicina de Lisboa |
| 4 | SAMS Lisbon | 10 | Comissão de Ética para a Saúde do Serviço de Asistência Médico-Social (SAMS) |
| 5 | Western Lisbon Hospital Center | 9 | Comissão de Ética para a Saúde do Centro Hospitalar de Lisboa Ocidental, EPE |
| 6 | University Hospital Center of Central Lisbon | 8 | Comissão de Ética para a Saúde do Centro Hospitalar de Lisboa Central, EPE |
| 7 | University Hospital Center of Coimbra | 8 | Comissão de Ética para a Saúde do Centro Hospitalar e Universitário de Coimbra, EPE |
| 8 | ULS Guarda | 6 | Comissão de Ética para a Saúde da Unidade Local de Saúde da Guarda, EPE |
| 9 | IPO Coimbra | 5 | Comissão de Ética do IPO Coimbra Francisco Gentil, EPE |
| 10 | SESARAM | 5 | Comissão de Ética para a Saúde do SESARAM, EPARAM |
| 11 | Leiria Hospital Center | 4 | Comissão de Ética do Centro Hospitalar de Leiria, EPE |
| 12 | São João University Hospital Center | 4 | Comissão de Ética para a Saúde do Centro Hospitalar Universitário de São João / Faculdade de Medicina da Universidade do Porto |
| 13 | ULS Matosinhos | 3 | Comissão de Ética para a Saúde da Unidade Local de Saúde de Matosinhos, EPE |
| 14 | Garcia de Orta Hospital | 2 | Comissão de Ética para a Saúde do Hospital Garcia de Orta, EPE |
| 15 | Setúbal Hospital Center | 2 | Comissão de Ética para a Saúde do Centro Hospitalar de Setúbal, EPE |
| 16 | ULS Alto Minho | 2 | Comissão de Ética para a Saúde da Unidade Local de Saúde do Alto Minho, EPE |
| 17 | Tâmega e Sousa Hospital Center | 1 | Comissão de Ética para a Saúde do Centro Hospitalar Tâmega e Sousa, EPE |
| 18 | Hospital do Espírito Santo de Évora | 1 | Comissão de Ética do Hospital do Espírito Santo de Évora, EPE |
| 19 | Champalimaud Foundation | 0 | Comissão de Ética para a Saúde da Fundação Champalimaud |
| 20 | HSO Guimarães | 0 | Comissão de Ética para a Saúde do Hospital Senhora da Oliveira - Guimarães |
| 21 | Beatriz Ângelo Hospital | 0 | Comissão de Ética para a Saúde do Hospital Beatriz Ângelo |
| 22 | Entre Douro e Vouga Hospital Center | 0 | Comissão de Ética para a Saúde do Centro Hospitalar de Entre Douro e Vouga, EPE |
| 23 | Trás-os-Montes and Alto Douro Hospital Center | 0 | Comissão de Ética para a Saúde do Centro Hospitalar de Trás-os-Montes e Alto Douro, EPE |
| 24 | CUF Descubertas | 0 | Comissão de Ética da CUF Descobertas |
